# Comparison of systemic right ventricular function in transposition of the great arteries after atrial switch and congenitally corrected transposition of the great arteries

**DOI:** 10.1186/1532-429X-18-S1-P152

**Published:** 2016-01-27

**Authors:** Michael Morcos, Philip J Kilner, David J Sahn, Willem Helbing, Harold Litt, Emanuela Valsangiacomo-Buechel, Florence Sheehan

**Affiliations:** 1 grid.34477.330000000122986657Department of Medicine, University of Washington, Seattle, WA USA; 2 grid.439338.6Royal Brompton Hospital, London, United Kingdom; 3 grid.5288.70000000097585690Oregon Health and Science University, Portland, OR USA; 4 grid.5645.2000000040459992XDepartment of Pediatrics (Division of Cardiology) and Radiology, Erasmus Medical Center Sophia Children's Hospital, Rotterdam, Netherlands; 5 grid.25879.310000000419368972University of Pennsylvania, Philadelphia, PA USA; 6 grid.412341.1University of CHildren's Hospital, Zurich, Switzerland; 7 grid.34477.330000000122986657Department of Cardiology, University of Washington, Seattle, WA USA

## Background

In patients with transposition of the great arteries corrected by interatrial baffle (TGA) and those with congenitally corrected transposition of the great arteries (ccTGA) the right ventricle (RV) is subjected to systemic pressures and fails prematurely. We sought to further characterize the geometric, global and regional functional differences between these two groups.

## Methods

Using cardiac magnetic resonance imaging (MRI) the RV was reconstructed from manually traced borders of the ventricles, valves, and other anatomic landmarks of 25 patients with TGA, 17 patients with ccTGA, and 9 normal subjects. Global function was assessed by calculating RV ejection fraction (RVEF) as well as tricuspid annular plane systolic excursion (TAPSE). Regional wall motion was assessed in ten anatomic territories of the RV using the centersurface method.

## Results

The RV in TGA and ccTGA was more dilated, rounder, and had reduced global and regional function when compared to the normal RV. RVEF correlated better with transverse than longitudinal contraction. When the subgroups were compared, TGA patients had lower RVEF than ccTGA (29.7 ± 6.5% vs. 34.7 ± 7.4 %, p = 0.02), lower normalized TAPSE (0.097 ± 0.035 vs. 0.177 ± 0.044, p <0.01), and weaker basal segment contraction (image 1 and 2). However RV shape was similar in the two groups, and there was only a tendency towards a more dilated TGA RV (end diastolic volume index 145 ± 35 vs. 132 ± 41 mL/m^2^ in ccTGA, p= 0.09).

## Conclusions

As a result of the hemodynamic overload, the RVs in both TGA and ccTGA are dilated and rounder than normal. The weak correlation of RVEF with TAPSE may be attributable to increased reliance on transverse shortening. Furthermore, TGA and ccTGA RVs have distinct wall motion and remodeling patterns with the added insult of reduced basilar function in the TGA RV. The possibility of basilar scarring, either attributable to congenital differences between the two patient populations, the interatrial baffle, or the surgery itself, may account for the morphological and functional differences seen between the two groups with systemic RVs.

**Figure 1:**
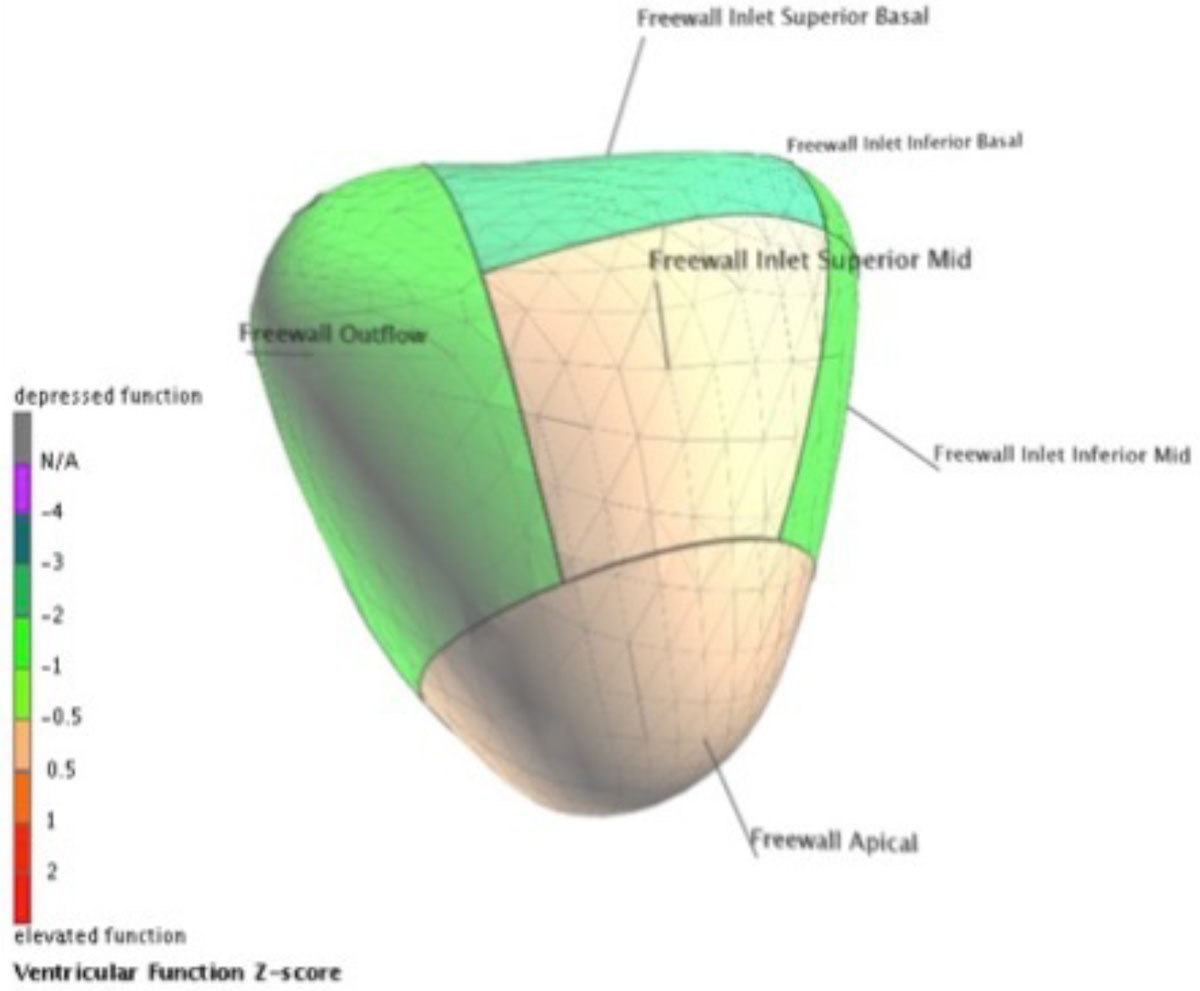
**Centersurface image of the RV free wall in a patient with ccTGA and dextrocardia**. Z scores are compared to normal subject and are color-coded as per figure legend.

**Figure 2:**
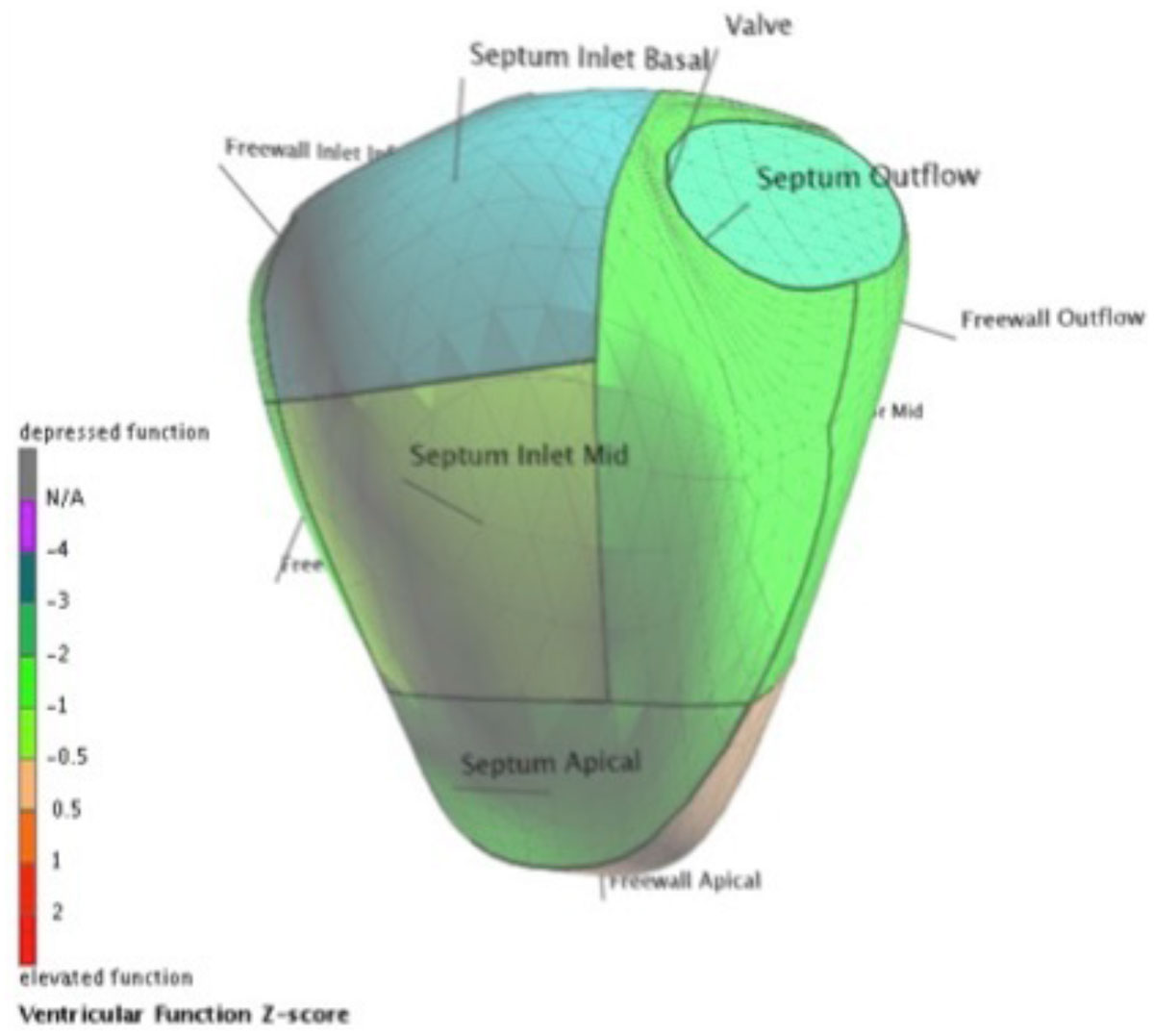
**Centersurface image of the RV septal wall in a patient with ccTGA and dextrocardia**. Z scores are compared to normal subject and are color-coded as per figure legend.

